# Design and Implementation of Improved Electronic Load Controller for Self-Excited Induction Generator for Rural Electrification

**DOI:** 10.1155/2015/340619

**Published:** 2015-12-13

**Authors:** C. Kathirvel, K. Porkumaran, S. Jaganathan

**Affiliations:** ^1^Sri Ramakrishna Engineering College, Coimbatore, Tamil Nadu, India; ^2^Dr. N. G. P. Institute of Technology, Coimbatore, Tamil Nadu, India

## Abstract

This paper offers an alternative technique, namely, Improved Electronic Load Controller (IELC), which is proposal to improve power quality, maintaining voltage at frequency desired level for rural electrification. The design and development of IELC are considered as microhydroenergy system. The proposed work aims to concentrate on the new schemes for rural electrification with the help of different kinds of hybrid energy systems. The objective of the proposed scheme is to maintain the speed of generation against fluctuating rural demand. The Electronic Load Controller (ELC) is used to connect and disconnect the dump load during the operation of the system, and which absorbs the load when consumer are not in active will enhance the lifestyle of the rural population and improve the living standards. Hydroelectricity is a promising option for electrification of remote villages in India. The conventional methods are not suitable to act as standalone system. Hence, the designing of a proper ELC is essential. The improved electronic load control performance tested with simulation at validated through hardware setup.

## 1. Introduction

The small scale microhydropower stations combine the advantages of hydropower with those of decentralized power generation without any disadvantages of large scale installations. Small scale hydropower has the advantages like economic distribution of energy, less environmental impacts compared with large hydrosystems, independence from imported fuels, and no need for expensive maintenance. Small scale hydropower can be used as decentralized energy systems for rural electrification.

Bassett and Potter have proposed a three-phase cage Induction Machine (IM) as a self-excited generator connected to the AC side of a voltage source. The generator is supposed to be driven by a low head unregulated shaft of microsystem. These systems intended to be applied in rural plants as a low-cost source of high quality AC sinusoidal regulated voltage with constant frequency [1].

Arrillaga and Watson proposed a static power conversion method from a Self-Excited Induction Generator [2].

Murthy et al. have proposed a simple and economical method for controlling a Self-Excited Induction Generator (SEIG) for standalone microhydropower generation [3].

Bhattacharya and Woodward have analyzed the performance of excitation balancing of Self-Excited Induction Generators (SEIG) supplying unbalanced loads. The additional drawbacks of SEIG are poor voltage regulation and require adjustable reactive power with varying load to maintain constant terminal voltage [4].

Bim et al. analyzed the performance of a voltage compensation based voltage regulator for Self-Excited Induction Generators (SEIG) supplying nonlinear loads [5].

Levy has presented importance of an Electronic Load Controller (ELC) for three-phase Self-Excited Induction Generators. The proposed generator was able to generate constant voltage and frequency, only if electrical load is maintained constant [6].

Wang and Su provided a comprehensive review of effect of long shunt and short shunt connections on permanent magnet generators, induction generators, synchronous generators, and doubly fed induction generators [7].

Rai et al. have presented the dynamic and steady state performance of a standalone Self-Excited Induction Generator with fuzzy logic controller (SEIG) using passive elements [8].

Singh et al. have presented a system based on a Self-Excited Induction Generator with shunt electronic converter to feed isolated three-phase and single phase linear or nonlinear loads [9].

Singh et al. have presented an Electronic Load Controller (IELC) based voltage and frequency regulator for an isolated asynchronous generator and demonstrated the improvements in the performance of Self-Excited Induction Generator [10].

Kuo and Wang have proposed the analysis of isolated self-induction generator feeding a rectifier load [11].

Wildi has proposed the voltage and frequency control of an autonomous Induction Generator (IG). A Voltage Source Inverter (VSI) with a Dump Load (DL) circuit is employed in its DC side. The IG frequency is controlled by keeping the VSI synchronous frequency constant [12].

Bansal has presented an overview about several solutions for standalone three-phase self-excited generators. A hybrid excited synchronous generator based on the two different types of excitation field is proposed [13].

Singh et al. have demonstrated the behavior of an Electronic Load Controller for Self-Excited Induction Generator under unbalanced grid voltage conditions. The phenomena are first analyzed theoretically as a function of the stator active and reactive instantaneous power exchange by the stator of the SEIG and the Grid Side Converter (GSC) [14].

Baroudi et al. have proposed new methods for power converter topologies consisting of a three-phase Self-Excited Induction Generator (SEIG) with STATCOM for feeding dynamic induction motor loads [15].

Mahato et al. analyzed the transient performance of a single phase self-regulated induction generator using a three-phase machine [16].

Singh [17] analyzed the performance of a self-excited six-phase induction generator for standalone renewable energy generation and modeled an efficient system.

Yokesh et al. (2010) proposed a voltage regulation scheme for Self-Excited Induction Generator for industry applications and analyzed the system with the help of different voltage and load conditions.

**Figure 1:**
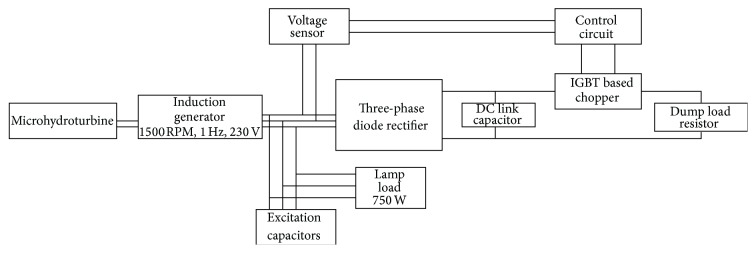
Block diagram of the proposed Improved Electronic Load Controller for microhydrosystem.

This paper considers a microhydrosystem at standalone mode. The microhydrosystems are consisting of a generating station, whose output power is less than 100 KW capacity. A squirrel cage induction motor can be used as a generator and capacitor bank of suitable rating is connected in both shunt or series and combination of both. It is required for supplying the VAR by the generator and the loads. The rotor is rotated at speed above the synchronous speed of the motor. The output voltage and frequency will be maintained within limits, under full load condition. When the consumer load is reduced, the excess load is consumed by the Improved Electronic Load Controller. The overall functional block diagram is shown in Figure 1.

## 2. Materials and Methods

### 2.1. Problem Formulation

**Figure 2:**
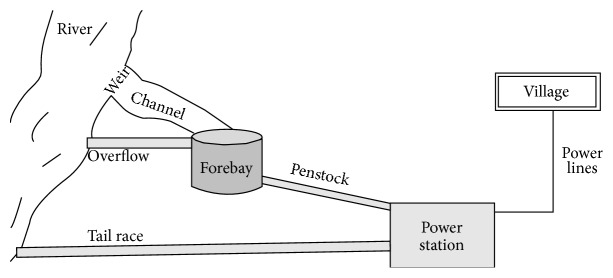
A typical microhydrosystem structure.

The microhydroenergy sources are available in plenty of places and usually seen in hilly areas where water flows as small rivers or streams. The schematic arrangement of microhydrosystem is shown in Figure 2; it is used to divert a small portion of this water by the way the power is generated. A fore bay is used to maintain the head constant; hence fore-bay should be ideally full all the time. The excess water overflows into the same river. Figure 2 shows the typical microhydrosystem structure.

The power generation of the proposed microhydrosystem is expressed in

(1) $$\begin{array}{c} P=\rho gQH, \end{array}$$

where *ρ* = water density, (kg/m^3^), *g* = gravitational acceleration, (m/s^2^), *Q* = discharge, (L/s), and *H* = head, (m).

In standalone microhydrosystem, An IELC is a solid state electronic device designed to regulate output power of a microhydropower system and also to regulate voltage to a desired level. The output voltage and frequency will be during full load condition and full load is considered throughout the operation of the consumer load. Support the load reduced and then the excess load is consumed by the Improved Electronic Load Controller (IELC):

(2) Power  generated=Power  consumed  by  load+Power  consumed  by  ELC.

Hence, it is the design of Electronic Load Controller for the satisfactory operation microhydrosystem.

### 2.2. Design Constraints

#### 2.2.1. Generator

**Figure 3:**
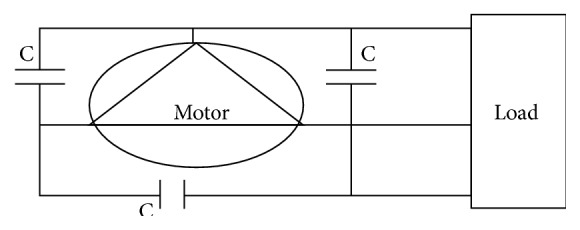
Three excitation capacitors for three-phase output.

The converting three-phase induction motor into a three-phase induction generator various designs are available. Here, the three-phase motor with three excitation capacitors for three-phase output can be used for this purpose as shown in Figure 3.

Normal single phase induction motors cannot be used as Self-Excited Single Phase Induction Generators (SEIG) because the modifications or additions are required to act as SEIG. Single phase induction machines of integral kW ratings are costwise high, which is compared with three-phase induction machine of equivalent size. It has been found that three-phase SEIG can be used for supplying single phase loads.

The motor is chosen taking into consideration the power output and the voltage rating. The rating of the machine is shown in Table 1.

#### 2.2.2. Design of Excitation Capacitor

The rating of the excitation capacitor is selected to produce the rated voltage at full load. The rating of the capacitor is chosen as per

(3) Apparent  power,  S=3·VL·IL,Active  power,  P=Scos⁡θ,Reactive  power  absorbed,  Q=S2−P2,Per  phase  reactive  power  needed,  q=Q3,Voltage  per  phase,  VP=VL3,Capacitive  current,  IC=qVP,Capacitive  reactance  per  phase,  XC=VPIC,Capacitance  per  phase,  C=12·πfXC,

based on the design of excitation capacitor rating 27 *μ*F/P for each phase of 3Φ Induction motor.

### 2.3. Electronic Load Controller

The voltage rating of the uncontrolled rectifier and chopper switch will be the same and dependent on the Root Mean Square (RMS) value of the AC input voltage and average value of the output DC voltage. The ratings of the various components of the proposed ELC are given as follows:

(4) $$\begin{array}{c} V_{DC}=3\sqrt{2}V_{LL/\pi }=\left(\;1.35\;\right)V_{LL}, \end{array}$$

where *V*_LL_ is the RMS value of the line-to-line voltage of SEIG. For the 750-watt SEIG, the line voltage is 230 V and the value of *V*_DC_ is given by

(5) $$\begin{array}{c} V_{DC}=\left(\;1.35\;\right)\times 230=310.5\;V. \end{array}$$

An overvoltage of 10% of the rated voltage is considered for transient conditions; hence the RMSAC input voltage which will be with a peak value is calculated using

(6) $$\begin{array}{c} V_{peak}=\sqrt{2}\times 253\;V=357.8\;VA. \end{array}$$

This peak voltage will appear across the components of ELC during the operation of the system. The current rating of the uncontrolled rectifier and chopper switch is decided by the active component of input AC current and calculated using

(7) $$\begin{array}{c} I_{AC}=\frac{P}{\sqrt{3}V_{LL}}, \end{array}$$

where *V*_LL_ is the RMS value of the SEIG terminal voltage and *P* is the power rating of SEIG. The active current of SEIG is calculated using

(8) $$\begin{array}{c} I_{AC}=\frac{750}{\sqrt{3}\times 230}=1.882. \end{array}$$

The three-phase uncontrolled rectifier draws approximately quasi-square current with the distortion factor of (3/*π* = .955). The input AC current of ELC is calculated using

(9) $$\begin{array}{c} I_{DAC}=\frac{I_{AC}}{0.955}=\frac{1.882}{0.955}. \end{array}$$

The Crest Factor (CF) of the AC current drawn by an uncontrolled rectifier with a capacitive filter varies from 1.4 to 2.0; hence, the AC input peak current may be calculated using

(10) $$\begin{array}{c} I_{peak}=2I_{DAC}=2\times 1.970=3.941. \end{array}$$

So the maximum voltage and peak current in the uncontrolled rectifier are 357.8 volts and 3.941 amperes, respectively. The rating of an uncontrolled rectifier and chopper switch is 600 V and 5 A, higher than the calculated values, respectively. The rating of dump load resistance is calculated by using

(11) $$\begin{array}{c} R_{D}=\frac{V_{DC}^{2}}{P_{rated}}. \end{array}$$

From this relation, the value of *R*_*D*_ is computed with

(12) $$\begin{array}{c} R_{D}=\frac{310.5^{2}}{750}=128.5\;ohms. \end{array}$$

The value of the DC link capacitance of the ELC is selected on the basis of the ripple factor. The relation between the values of DC link capacitance and Ripple Factor (RF) for a three-phase uncontrolled rectifier is given by

(13) $$\begin{array}{c} C=\frac{1}{12fR_{D}}\cdot 1+\frac{1}{\sqrt{2}RF}. \end{array}$$

Normally, 5% ripple factor is permitted in the average value of DC link voltage. The capacitance is calculated using the previous formula and hence the value of the capacitance is given by

(14) $$\begin{array}{c} C=\frac{1}{12\times 50\;128.5}\cdot \left(\;1+\frac{1}{\sqrt{2}\times 0.05}\;\right)=196.39\;\mu F. \end{array}$$

The entire ratings of the components of proposed ELC are given in Table 2.

## 3. Results and Discussion

**Figure 4:**
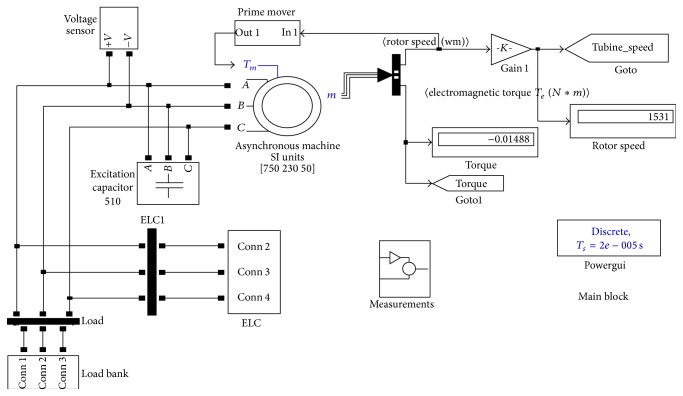
Main block of the proposed system.

**Figure 5:**
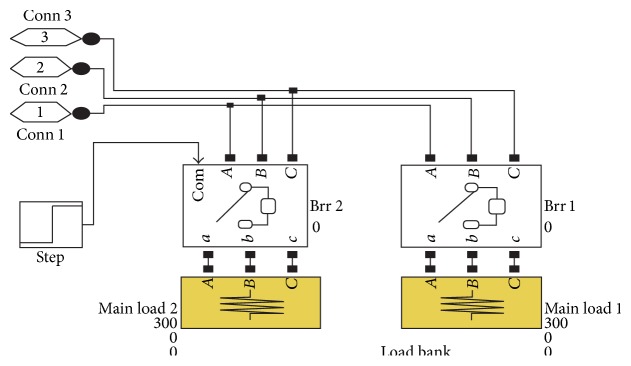
Load bank.

**Figure 6:**
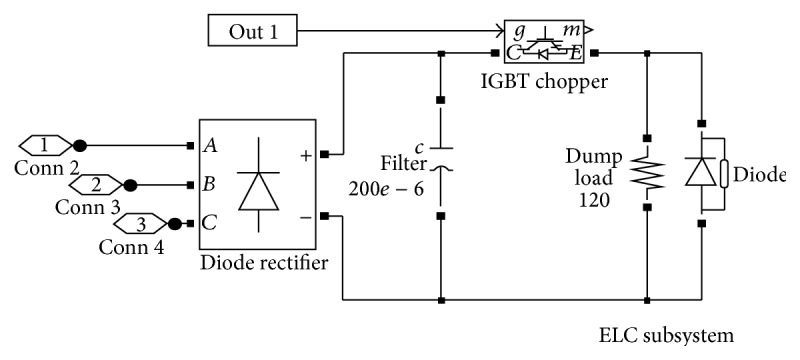
IELC block.

The various blocks of the simulation circuits of the entire system are given in Figures 4, 5, and 6. The simulation studies on performance of SEIG with IELC were illustrated in Figure 4. The Simulation is carried out using MATLAB/Version.

Figure 4 gives exact model for the proposed system. The number of loads all which is connected in IELC is clearly described in Figure 4. The complexity of the system is reduced by selecting advanced measurement blocks. The load levels are depending upon the compensation; the compensating voltage levels are described in the design considerations. The loads are controlled through the effective design of IELC. Once the compensation analysis is over in successful manner, the exclusive voltage is consumed by IELC. The input power SEIG is maintained as a constant value corresponding with the varying consumer loads. The load bank circuit is illustrated in Figure 5.

Figure 6 shows the IELC block. The IELC is to design so as to maintain the desired load level which enables the voltage and frequency in constant level. This configuration is used to make the SEIG connected in turbine system. The Improved Electronic Load Controller is an electronic device that maintains a constant electrical load against changing loads in the generating station.

**Figure 7:**
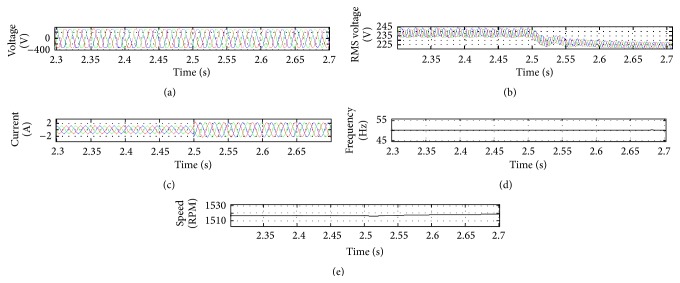
(a) Simulation waveforms of output voltage. (b) Output RMS voltage. (c) Output current. (d) Frequency. (e) Speed.

**Figure 8:**
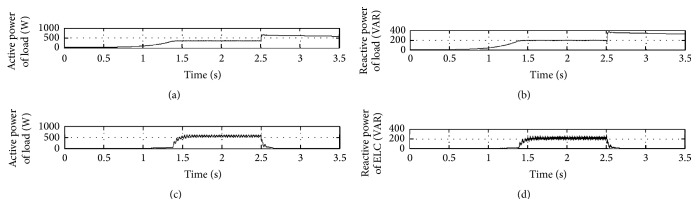
(a) Active power of load. (b) Reactive power of load. (c) Active power of ELC. (d) Reactive power of ELC.

The output waveforms are shown in Figures 7 and 8. It can be observed that the initial load of 300 milliwatts is changed to 600 watts at 2.5 s and the voltage and frequency vary only slightly as it is within limits.

The simulation output reveals that the voltage and frequency remain constant for varying load conditions.  Figure 7 shows the waveforms of the 3-phase rms voltage, 3-phase instantaneous current, frequency, and speed which is measured in rpm. The hardware experimental setup figures whenever the load changes due to the consumer loads.

The IELC is bringing the regulation of voltage and maintaining the SEIG speed at a constant value. The constant value of speed is achieved by SEIG; this is because IELC current increases and decreased against the load deviation. The active load power, reactive load power, the active power, and reactive power are illustrated in Figure 8.

Figure 8 shows the simulation waveforms of active power of load, reactive power of load, active power of ELC, and reactive power of ELC.

From the results, the simultaneous performance of proposed system shows that the microhydroschemes combined with improved load controller are produced. The good is shown. The constant power and sustainable power for rural electrification are added advantage in connection with the hardware implementation which is done based on simulation performance.

**Figure 9:**
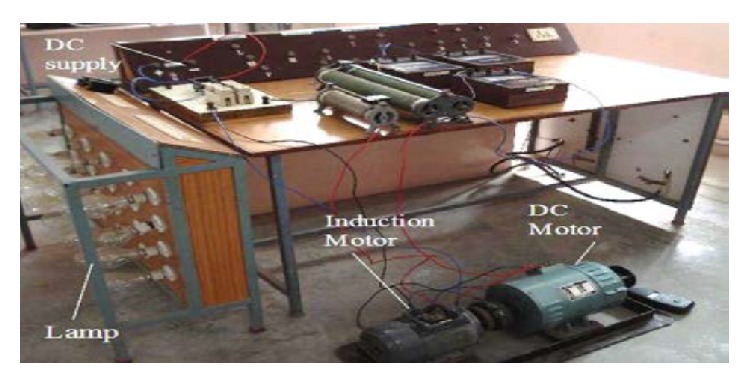
Hardware experimental setup.

*Experimental Setup*. The experimental setup consists of a conventional three-phase induction motor coupled to a separately excited DC motor. It can be observed that when the initial load of 300 W is changed from 600 W at 2.5 seconds, the active and reactive power consumption of the load increase and those of the ELC decrease.  Figure 9 shows the hardware experimental setup.

The generator was tested without connecting the ELC and control circuit by connecting three 20 microfarad capacitors in star across the stator output. The test results are tabulated in Table 3.

**Figure 10:**
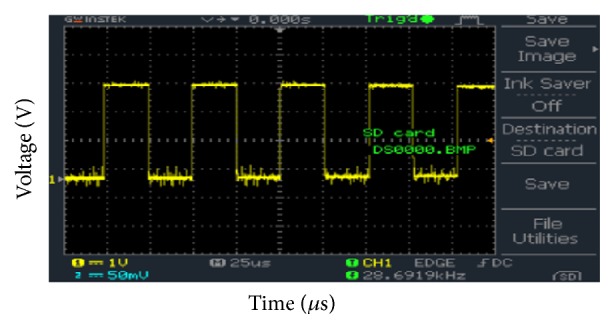
CCP1 pin output of PIC.

The hardware experimental results are obtained using GwINSTEK Digital Storage Oscilloscope (DSO).  Figure 10 shows the output of CCP1 pin of the microcontroller. The frequency was 19.53 kHz and duty cycle was 50%. It can be observed that the peak voltage of the PWM signal is 3.2 V.

**Figure 11:**
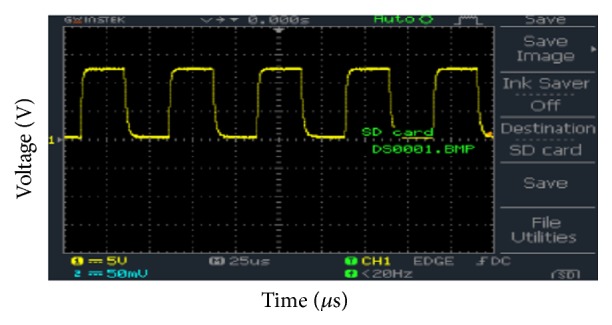
Optocoupler output.

Figure 11 shows the output of the optocoupler. The optocoupler is used to isolate the high power circuit from the low power circuit so that if some failure occurs in the high power circuit, this will not be propagated to the low power control circuit. The input frequency was 19.53 kHz and duty cycle was 50%. It can be observed that the peak voltage of the PWM signal is 12.5 V.

**Figure 12:**
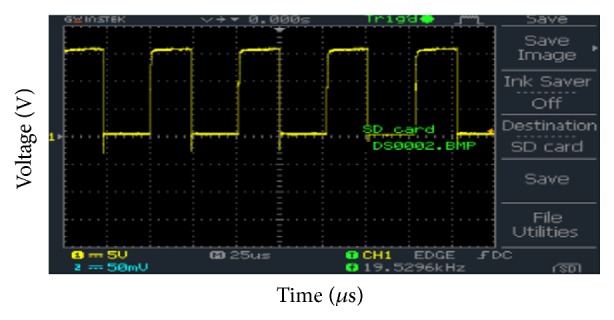
Gate emitter voltage of IGBT.

Figure 12 shows the voltage across the dump resistor load when RC snubber across the IGBT is connected. The input frequency is 19.53 kHz and duty cycle was 50%.

**Figure 13:**
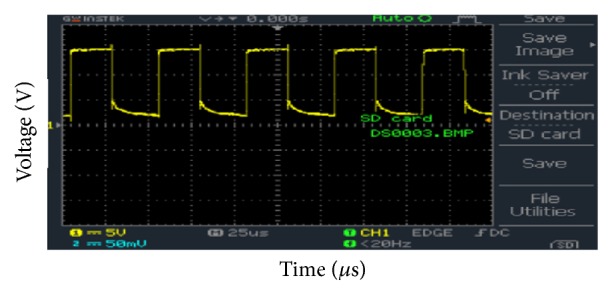
Voltages across 50 ohm resistor with snubber.

Figure 13 shows the voltage across 50 ohm resistor with snubber.

**Figure 14:**
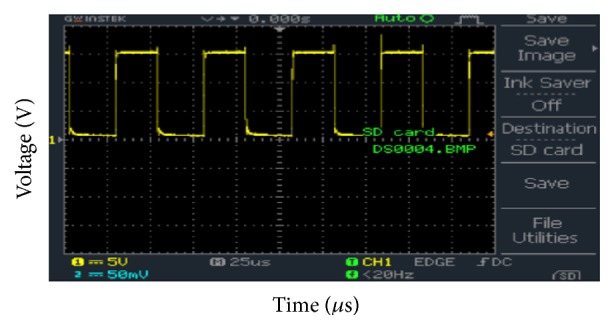
Voltage across 50 ohm resistor without snubber.

Figure 14 shows the voltage across the dump resistor load without the RC snubber. The input frequency was 19.53 kHz and duty cycle 50%. It can be observed that the steady state peak voltage of the PWM signal is approximately 15 V and the transient peak voltage is higher than that with the snubber. The supply voltage given to the dump load was 15 V.

## 4. Conclusion

The proposed microhydrosystem was designed and implemented with the IELC. The simulation results revealed that the voltage and frequency remain within limits during the load changes. The hardware experimental setup was fabricated by using a DC motor as the prime mover. The IELC and control circuits were designed and fabricated. The hardware experimental setup was first tested by giving 15 V regulated power supply instead of the rectified output supply from the generator. Then, it was tested with the output voltage of the induction generator through the diode rectifier. The IELC was operating properly by consuming the excess voltage during the load level which is below the nominal level. The IELC system is cost-effective and is easily constructible in rural areas. Compared to existing methods of ELC, the IELC gives prominent merits. The voltage and frequency maintained constancy through the operation. So power quality is improved.

## Figures and Tables

**Table 1:** Induction Machine ratings.

Parameter	Specification
Motor type	Squirrel cage induction motor
Phase	3 phases
Line voltage	230 V
Rated speed	1485 RPM
Horsepower	1 H.P

**Table 2:** Ratings of the components of proposed ELC.

Power rating of motor (W)	Voltage rating of rectifier (V)	Current rating of rectifier (A)	Voltage rating of chopper switch (V)	Current rating of chopper switch (A)	Rating of dump load (*Ω*)	Rating of DC filtering capacitor (*μ*F)

750	600	5	600	5	125	200

**Table 3:** Test results of generator.

Load	Speed (RPM)	Phase voltage (V)	Frequency (Hz)
No load	1505	475	49.9
205 ohms per phase in star	1430	255	47
